# Crystal structure of bis­[2-*tert*-but­oxy-6-fluoro-3-(pyridin-2-yl-κ*N*)pyridin-4-yl-κ*C* ^4^](pentane-2,4-dionato-κ^2^ *O*,*O*′)iridium(III)

**DOI:** 10.1107/S1600536814022934

**Published:** 2014-10-29

**Authors:** Ki-Min Park, Youngjin Kang

**Affiliations:** a Research Institute of Natural Science, Gyeongsang National University, Jinju, 660-701, South Korea; b Division of Science Education and Department of Chemistry, Kangwon National, University, Chuncheon 220-701, South Korea

**Keywords:** crystal structure, iridium(III), C_2_N_2_O_2_ coordination set

## Abstract

The title mol­ecule, [Ir(C_14_H_14_FN_2_O)_2_(C_5_H_7_O_2_)], is located on a twofold rotation axis, which passes through the Ir^III^ atom and the central C atom of the pentane-2,4-dionate anion. The Ir^III^ atom adopts a distorted octa­hedral coordination geometry, being *C*,*N*-chelated by two 2-*tert*-but­oxy-6-fluoro-3-(pyridin-2-yl)pyridin-4-yl ligands and *O*,*O′*-chelated by the pentane-2,4-dionato ligand. The bipyridinate ligands, which are perpendicular to each other [dihedral angle between the two least-squares planes = 89.95 (5)°], are arranged in a *cis*-*C*,*C′* and *trans*-*N*,*N′* fashion relative to the central metal cation. Intra­molecular C—H⋯O and C—H⋯N hydrogen bonds and inter­molecular C—H⋯F hydrogen bonds as well as π—π inter­actions between neighbouring pyridine rings [centroid–centroid distance 3.680 (1) Å] contribute to the stabilization of the mol­ecular and crystal structure, respectively.

## Abstract 2

The Ir^III^ atom in the title mol­ecule adopts a distorted octa­hedral coordination sphere, being *C*,*N*-chelated by two main 2-tert-but­oxy-6-fluoro-3-(pyridine-2-yl)pyridine-4-yl ligands and *O*,*O′*-chelated by one ancillary pentane-2,4-dionato ligand.

## Chemical context   

Iridium(III) compounds with fluorinated main dipyridyl ligands have attracted much attention due to their colour purity and high external quantum efficiency in organic light-emitting diodes (Lee *et al.*, 2009 ▶; Park *et al.*, 2013 ▶). In particular, heteroleptic Ir^III^ compounds have many advantages such as easy tuning of emission energies and photophysical properties by modification of the ancillary ligands (Oh *et al.*, 2013 ▶). Herein, we report the results of the crystal-structure determination of an iridium(III) compound, [Ir(C_14_H_14_FN_2_O)_2_(C_5_H_7_O_2_)], with acetylacetonate (acac, *O,O′*) as an ancillary ligand.
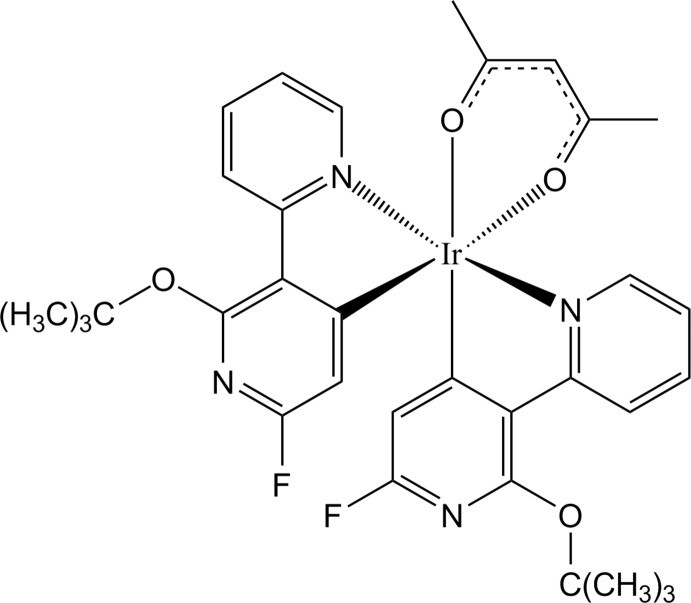


## Structural commentary   

**Figure 1:**
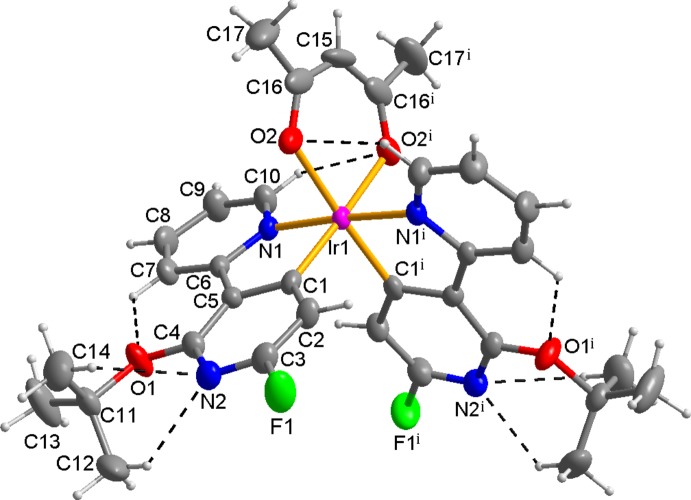
View of the mol­ecular structure of the title compound, with the atom-numbering scheme. Displacement ellipsoids are drawn at the 50% probability level; dashed lines represent intra­molecular C—H⋯O and C—H⋯N hydrogen bonds [Symmetry code: (i) − *x*, *y*, 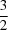 − *z*].

The mol­ecular structure of the title compound, Fig. 1 ▶, is generated by twofold rotation symmetry. The twofold rotation axis passes through the Ir^III^ atom and the central C atom (C15) of the acetyl­acetonate ligand. Therefore, the asymmetric unit consists of one Ir(III) atom on Wyckoff position 4*e*, one half of the acetyl­acetonate anion and one 2-*tert*-but­oxy-6-fluoro-3-(pyridin-2-yl)pyridin-4-yl ligand. The Ir^III^ atom is six-coord­inated by the two main *C*,*N*-bidentate ligands and one ancillary *O*,*O′*-bidentate ligand, forming a distorted octa­hedral coordination sphere due to the narrow ligand bite angles, which range from 80.36 (7) to 88.65 (8)°. The *C*,*N*-bidentate ligands, which are perpendicular to each other [dihedral angle between the least-squares planes = 89.95 (5)°], are arranged in a *cis*-*C*,*C′* and *trans*-*N*,*N′* fashion. The Ir—C bond length of 1.9760 (19) Å is shorter than the Ir—N bond length of 2.0344 (16) Å due to the electronegative fluorine substituent (Table 1 ▶). The Ir—C, Ir—N, and Ir—O bond lengths are in normal ranges as reported for similar Ir^III^ compounds, *e.g.* [Ir(dfpypy)_2_(acac); dfpypy is a difuorinated bi­pyridine] (Kang *et al.*, 2013 ▶) or Ir(2′,6′-bis­(2-meth­oxy­eth­oxy)-2,3′-bipyridinato-*N*,*C′*)(picolinate) (Frey *et al.*, 2014 ▶). Within the *C*,*N-*bidentate ligand of the title compound, the two pyridine rings are approximately co-planar, with a dihedral angle between the rings of 5.77 (9)°.

## Supra­molecular features   

**Figure 2:**
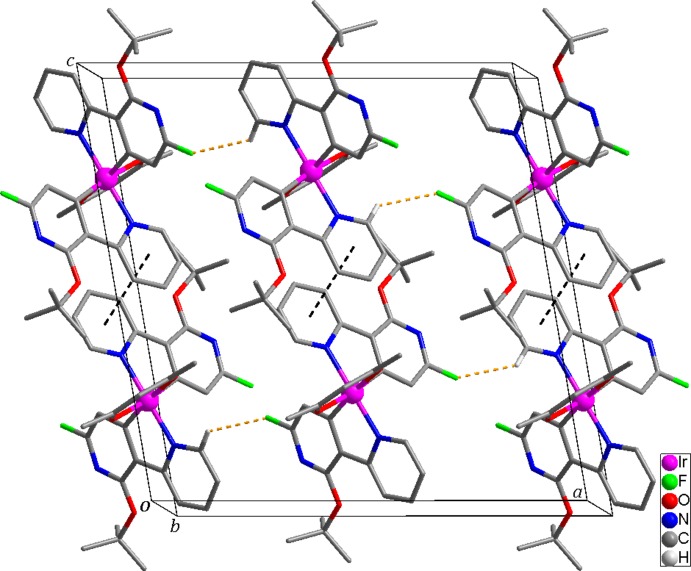
Packing plot of the mol­ecular components in the title compound. Yellow and black dashed lines represent inter­molecular C—H⋯F and π–π stacking inter­actions, respectively. H atoms not involved in inter­molecular inter­actions have been omitted for clarity.

The mol­ecular structure is stabilized by weak intra­molecular C—H⋯O and C—H⋯N hydrogen bonds (Table 2 ▶). Inter­molecular C—H⋯F hydrogen bonds and π—π inter­actions [*Cg*1—*Cg*1^iii^ = 3.680 (1) Å, *Cg*1 is the centroid of the N1, C6–C10 ring, symmetry code: (iii) −*x*, 1 − *y*, 2 − *z*] contribute to the stabilization of the crystal structure (Fig. 2 ▶).

## Synthesis and crystallization   

The title compound was synthesized according to a previous report (Oh *et al.*, 2013 ▶). Yellow single crystals were obtained by slow evaporation from a di­chloro­methane/hexane solution.

## Refinement   

Crystal data, data collection and crystal structure refinement details are summarized in Table 3 ▶. All H atoms were positioned geometrically and refined using a riding model, with *d*(C—H) = 0.95 Å, *U*_iso_(H) = 1.2*U*_eq_(C) for C*sp* ^2^ H atoms, and 0.98 Å, *U*_iso_(H) = 1.5*U*_eq_(C) for methyl protons.

## Supplementary Material

Crystal structure: contains datablock(s) I, New_Global_Publ_Block. DOI: 10.1107/S1600536814022934/wm5075sup1.cif

Structure factors: contains datablock(s) I. DOI: 10.1107/S1600536814022934/wm5075Isup2.hkl

CCDC reference: 1029929

Additional supporting information:  crystallographic information; 3D view; checkCIF report

## Figures and Tables

**Table 1:** Selected bond lengths ()

Ir1C1	1.9760(19)	Ir1O2	2.1393(15)
Ir1N1	2.0344(16)		

**Table 2:** Hydrogen-bond geometry (, )

*D*H*A*	*D*H	H*A*	*D* *A*	*D*H*A*
C7H7O1	0.95	2.27	2.870(2)	120
C10H10O2^i^	0.95	2.48	3.089(2)	122
C10H10F1^ii^	0.95	2.41	3.055(2)	125
C12H12*C*N2	0.98	2.29	2.927(3)	122
C14H14*B*N2	0.98	2.59	3.153(3)	116

Symmetry codes: (i) 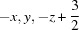; (ii) 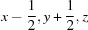.

**Table 3:** Experimental details

Crystal data	
Chemical formula	[Ir(C_14_H_14_FN_2_O)_2_(C_5_H_7_O_2_)]
*M*_r_	781.85
Crystal system, space group	Monoclinic, *C*2/*c*
Temperature (K)	173
*a*, *b*, *c* ()	16.9404(12), 10.7783(7), 17.2561(11)
()	100.001(1)
*V* (^3^)	3102.9(4)
*Z*	4
Radiation type	Mo *K*
(mm^1^)	4.36
Crystal size (mm)	0.16 0.12 0.09

Data collection	
Diffractometer	Bruker APEXII CCD
Absorption correction	Multi-scan (*SADABS*; Sheldrick, 1996 ▶)
*T*_min_, *T*_max_	0.537, 0.687
No. of measured, independent and observed [*I* > 2(*I*)] reflections	15125, 3881, 3717
*R*_int_	0.024
(sin /)_max_ (^1^)	0.668

Refinement	
*R*[*F* ^2^ > 2(*F* ^2^)], *wR*(*F* ^2^), *S*	0.017, 0.039, 1.01
No. of reflections	3881
No. of parameters	200
H-atom treatment	H-atom parameters constrained
_max_, _min_ (e ^3^)	0.48, 0.59

Computer programs: *APEX2* and *SAINT* (Bruker, 2006 ▶), *SHELXS97*, *SHELXL97* and *SHELXTL* (Sheldrick, 2008 ▶), *DIAMOND* (Brandenburg, 2005 ▶) and *publCIF* (Westrip, 2010 ▶).

## supplementary crystallographic information

### Crystal data

**Table:**

[Ir(C_14_H_14_FN_2_O)_2_(C_5_H_7_O_2_)]	*F*(000) = 1552
*M**_r_* = 781.85	*D*_x_ = 1.674 Mg m^−^^3^
Monoclinic, *C*2/*c*	Mo *K*α radiation, λ = 0.71073 Å
Hall symbol: -C 2yc	Cell parameters from 3721 reflections
*a* = 16.9404 (12) Å	θ = 2.3–28.3°
*b* = 10.7783 (7) Å	µ = 4.36 mm^−^^1^
*c* = 17.2561 (11) Å	*T* = 173 K
β = 100.001 (1)°	Block, yellow
*V* = 3102.9 (4) Å^3^	0.16 × 0.12 × 0.09 mm
*Z* = 4	

### Data collection

**Table:**

Bruker APEXII CCD diffractometer	3881 independent reflections
Radiation source: fine-focus sealed tube	3717 reflections with *I* > 2σ(*I*)
Graphite monochromator	*R*_int_ = 0.024
φ and ω scans	θ_max_ = 28.3°, θ_min_ = 2.3°
Absorption correction: multi-scan (*SADABS*; Sheldrick, 1996)	*h* = −22→21
*T*_min_ = 0.537, *T*_max_ = 0.687	*k* = −14→14
15125 measured reflections	*l* = −15→23

### Refinement

**Table:**

Refinement on *F*^2^	Primary atom site location: structure-invariant direct methods
Least-squares matrix: full	Secondary atom site location: difference Fourier map
*R*[*F*^2^ > 2σ(*F*^2^)] = 0.017	Hydrogen site location: inferred from neighbouring sites
*wR*(*F*^2^) = 0.039	H-atom parameters constrained
*S* = 1.01	*w* = 1/[σ^2^(*F*_o_^2^) + (0.0163*P*)^2^ + 5.6671*P*] where *P* = (*F*_o_^2^ + 2*F*_c_^2^)/3
3881 reflections	(Δ/σ)_max_ = 0.001
200 parameters	Δρ_max_ = 0.48 e Å^−^^3^
0 restraints	Δρ_min_ = −0.59 e Å^−^^3^

### Special details

**Table:**

Geometry. All e.s.d.'s (except the e.s.d. in the dihedral angle between two l.s. planes) are estimated using the full covariance matrix. The cell e.s.d.'s are taken into account individually in the estimation of e.s.d.'s in distances, angles and torsion angles; correlations between e.s.d.'s in cell parameters are only used when they are defined by crystal symmetry. An approximate (isotropic) treatment of cell e.s.d.'s is used for estimating e.s.d.'s involving l.s. planes.
Refinement. Refinement of *F*^2^ against ALL reflections. The weighted *R*-factor *wR* and goodness of fit *S* are based on *F*^2^, conventional *R*-factors *R* are based on *F*, with *F* set to zero for negative *F*^2^. The threshold expression of *F*^2^ > σ(*F*^2^) is used only for calculating *R*-factors(gt) *etc*. and is not relevant to the choice of reflections for refinement. *R*-factors based on *F*^2^ are statistically about twice as large as those based on *F*, and *R*- factors based on ALL data will be even larger.

### Fractional atomic coordinates and isotropic or equivalent isotropic displacement parameters (Å^2^)

**Table:**

	*x*	*y*	*z*	*U*_iso_*/*U*_eq_	
Ir1	0.0000	0.424606 (10)	0.7500	0.01166 (4)	
F1	0.22407 (8)	0.06652 (13)	0.79551 (8)	0.0273 (3)	
O1	0.08930 (9)	0.14456 (15)	0.99685 (8)	0.0214 (3)	
N1	−0.04307 (9)	0.41821 (15)	0.85288 (10)	0.0135 (3)	
N2	0.15918 (10)	0.10711 (16)	0.89537 (10)	0.0180 (4)	
C1	0.06958 (11)	0.29314 (18)	0.80481 (11)	0.0126 (4)	
C2	0.12693 (11)	0.2228 (2)	0.77450 (12)	0.0169 (4)	
H2	0.1371	0.2354	0.7226	0.020*	
C3	0.16737 (11)	0.1357 (2)	0.82308 (12)	0.0180 (4)	
C4	0.10416 (11)	0.17160 (19)	0.92486 (12)	0.0162 (4)	
C5	0.05807 (10)	0.26689 (18)	0.88253 (11)	0.0127 (4)	
C6	−0.00488 (11)	0.33982 (18)	0.90962 (11)	0.0129 (4)	
C7	−0.02889 (12)	0.33774 (19)	0.98317 (11)	0.0160 (4)	
H7	−0.0015	0.2861	1.0237	0.019*	
C8	−0.09224 (12)	0.41045 (19)	0.99727 (12)	0.0190 (4)	
H8	−0.1080	0.4099	1.0475	0.023*	
C9	−0.13243 (12)	0.4840 (2)	0.93726 (12)	0.0205 (4)	
H9	−0.1776	0.5317	0.9449	0.025*	
C10	−0.10572 (12)	0.4864 (2)	0.86643 (12)	0.0179 (4)	
H10	−0.1325	0.5382	0.8256	0.021*	
C11	0.13969 (13)	0.06280 (19)	1.05388 (12)	0.0189 (4)	
C12	0.14509 (17)	−0.0675 (2)	1.02218 (16)	0.0335 (6)	
H12A	0.0912	−0.1032	1.0093	0.050*	
H12B	0.1781	−0.1189	1.0621	0.050*	
H12C	0.1695	−0.0647	0.9747	0.050*	
C13	0.09314 (16)	0.0622 (3)	1.12145 (15)	0.0358 (6)	
H13A	0.0397	0.0271	1.1035	0.054*	
H13B	0.0879	0.1474	1.1398	0.054*	
H13C	0.1217	0.0118	1.1647	0.054*	
C14	0.22125 (14)	0.1217 (3)	1.07830 (14)	0.0307 (5)	
H14A	0.2148	0.2057	1.0979	0.046*	
H14B	0.2487	0.1256	1.0329	0.046*	
H14C	0.2530	0.0718	1.1199	0.046*	
O2	0.08159 (9)	0.56660 (14)	0.79971 (9)	0.0209 (3)	
C15	0.0000	0.7398 (3)	0.7500	0.0318 (8)	
H15	0.0000	0.8280	0.7500	0.038*	
C16	0.06984 (15)	0.6828 (2)	0.78927 (13)	0.0244 (5)	
C17	0.14050 (18)	0.7636 (3)	0.82222 (15)	0.0390 (6)	
H17A	0.1846	0.7114	0.8482	0.059*	
H17B	0.1248	0.8218	0.8605	0.059*	
H17C	0.1579	0.8100	0.7794	0.059*	

### Atomic displacement parameters (Å^2^)

**Table:**

	*U*^11^	*U*^22^	*U*^33^	*U*^12^	*U*^13^	*U*^23^
Ir1	0.01340 (5)	0.01297 (5)	0.00844 (5)	0.000	0.00137 (4)	0.000
F1	0.0230 (6)	0.0369 (8)	0.0223 (7)	0.0164 (6)	0.0047 (5)	−0.0021 (6)
O1	0.0240 (7)	0.0250 (8)	0.0158 (7)	0.0097 (6)	0.0052 (6)	0.0096 (6)
N1	0.0140 (7)	0.0146 (8)	0.0117 (8)	−0.0011 (6)	0.0017 (6)	−0.0012 (6)
N2	0.0173 (8)	0.0188 (9)	0.0170 (9)	0.0019 (7)	0.0003 (7)	−0.0005 (7)
C1	0.0107 (8)	0.0140 (9)	0.0120 (9)	−0.0023 (7)	−0.0014 (7)	−0.0026 (7)
C2	0.0151 (9)	0.0228 (10)	0.0126 (9)	0.0011 (8)	0.0019 (7)	−0.0020 (8)
C3	0.0133 (9)	0.0212 (11)	0.0188 (10)	0.0029 (8)	0.0009 (7)	−0.0048 (8)
C4	0.0159 (9)	0.0172 (10)	0.0146 (10)	−0.0018 (8)	0.0003 (7)	0.0007 (8)
C5	0.0118 (8)	0.0147 (9)	0.0112 (9)	−0.0015 (7)	0.0007 (7)	−0.0005 (7)
C6	0.0138 (8)	0.0123 (9)	0.0119 (9)	−0.0018 (7)	0.0001 (7)	−0.0003 (7)
C7	0.0190 (9)	0.0161 (10)	0.0128 (10)	−0.0001 (8)	0.0028 (7)	0.0020 (8)
C8	0.0219 (10)	0.0219 (11)	0.0147 (10)	−0.0009 (8)	0.0072 (8)	−0.0003 (8)
C9	0.0199 (10)	0.0243 (11)	0.0188 (10)	0.0061 (9)	0.0072 (8)	−0.0002 (9)
C10	0.0183 (9)	0.0204 (10)	0.0151 (10)	0.0046 (8)	0.0033 (8)	0.0023 (8)
C11	0.0226 (10)	0.0180 (10)	0.0143 (10)	0.0030 (8)	−0.0018 (8)	0.0062 (8)
C12	0.0483 (15)	0.0170 (11)	0.0309 (14)	−0.0015 (11)	−0.0051 (11)	0.0037 (10)
C13	0.0379 (14)	0.0486 (17)	0.0223 (12)	0.0122 (12)	0.0091 (10)	0.0177 (11)
C14	0.0304 (12)	0.0350 (13)	0.0236 (12)	−0.0092 (11)	−0.0041 (10)	−0.0018 (10)
O2	0.0287 (8)	0.0202 (8)	0.0137 (7)	−0.0078 (6)	0.0034 (6)	−0.0029 (6)
C15	0.055 (2)	0.0143 (15)	0.0311 (19)	0.000	0.0203 (16)	0.000
C16	0.0433 (13)	0.0193 (11)	0.0147 (10)	−0.0088 (10)	0.0161 (9)	−0.0042 (8)
C17	0.0612 (17)	0.0295 (14)	0.0271 (14)	−0.0228 (13)	0.0095 (12)	−0.0064 (11)

### Geometric parameters (Å, º)

**Table:**

Ir1—C1^i^	1.9760 (19)	C9—C10	1.375 (3)
Ir1—C1	1.9760 (19)	C9—H9	0.9500
Ir1—N1^i^	2.0344 (16)	C10—H10	0.9500
Ir1—N1	2.0344 (16)	C11—C14	1.512 (3)
Ir1—O2	2.1393 (15)	C11—C12	1.516 (3)
Ir1—O2^i^	2.1393 (14)	C11—C13	1.517 (3)
F1—C3	1.365 (2)	C12—H12A	0.9800
O1—C4	1.342 (2)	C12—H12B	0.9800
O1—C11	1.477 (2)	C12—H12C	0.9800
N1—C10	1.345 (3)	C13—H13A	0.9800
N1—C6	1.368 (2)	C13—H13B	0.9800
N2—C3	1.315 (3)	C13—H13C	0.9800
N2—C4	1.333 (3)	C14—H14A	0.9800
C1—C2	1.403 (3)	C14—H14B	0.9800
C1—C5	1.417 (3)	C14—H14C	0.9800
C2—C3	1.362 (3)	O2—C16	1.276 (3)
C2—H2	0.9500	C15—C16	1.400 (3)
C4—C5	1.414 (3)	C15—C16^i^	1.400 (3)
C5—C6	1.465 (3)	C15—H15	0.9500
C6—C7	1.399 (3)	C16—C17	1.509 (3)
C7—C8	1.384 (3)	C17—H17A	0.9800
C7—H7	0.9500	C17—H17B	0.9800
C8—C9	1.385 (3)	C17—H17C	0.9800
C8—H8	0.9500		
			
C1^i^—Ir1—C1	88.37 (10)	C10—C9—C8	118.72 (19)
C1^i^—Ir1—N1^i^	80.36 (7)	C10—C9—H9	120.6
C1—Ir1—N1^i^	96.83 (7)	C8—C9—H9	120.6
C1^i^—Ir1—N1	96.83 (7)	N1—C10—C9	122.37 (19)
C1—Ir1—N1	80.36 (7)	N1—C10—H10	118.8
N1^i^—Ir1—N1	176.12 (9)	C9—C10—H10	118.8
C1^i^—Ir1—O2	174.32 (7)	O1—C11—C14	109.26 (17)
C1—Ir1—O2	91.77 (7)	O1—C11—C12	112.12 (18)
N1^i^—Ir1—O2	93.98 (6)	C14—C11—C12	112.3 (2)
N1—Ir1—O2	88.80 (6)	O1—C11—C13	101.36 (17)
C1^i^—Ir1—O2^i^	91.77 (7)	C14—C11—C13	111.1 (2)
C1—Ir1—O2^i^	174.32 (7)	C12—C11—C13	110.2 (2)
N1^i^—Ir1—O2^i^	88.80 (6)	C11—C12—H12A	109.5
N1—Ir1—O2^i^	93.98 (6)	C11—C12—H12B	109.5
O2—Ir1—O2^i^	88.65 (8)	H12A—C12—H12B	109.5
C4—O1—C11	124.52 (16)	C11—C12—H12C	109.5
C10—N1—C6	120.19 (17)	H12A—C12—H12C	109.5
C10—N1—Ir1	123.15 (14)	H12B—C12—H12C	109.5
C6—N1—Ir1	116.67 (12)	C11—C13—H13A	109.5
C3—N2—C4	115.84 (18)	C11—C13—H13B	109.5
C2—C1—C5	117.59 (18)	H13A—C13—H13B	109.5
C2—C1—Ir1	127.09 (15)	C11—C13—H13C	109.5
C5—C1—Ir1	115.31 (13)	H13A—C13—H13C	109.5
C3—C2—C1	116.74 (18)	H13B—C13—H13C	109.5
C3—C2—H2	121.6	C11—C14—H14A	109.5
C1—C2—H2	121.6	C11—C14—H14B	109.5
N2—C3—C2	128.36 (19)	H14A—C14—H14B	109.5
N2—C3—F1	113.52 (18)	C11—C14—H14C	109.5
C2—C3—F1	118.12 (18)	H14A—C14—H14C	109.5
N2—C4—O1	119.76 (18)	H14B—C14—H14C	109.5
N2—C4—C5	122.80 (18)	C16—O2—Ir1	124.86 (15)
O1—C4—C5	117.42 (17)	C16—C15—C16^i^	127.9 (3)
C4—C5—C1	118.65 (17)	C16—C15—H15	116.1
C4—C5—C6	126.30 (17)	C16^i^—C15—H15	116.1
C1—C5—C6	114.98 (17)	O2—C16—C15	126.7 (2)
N1—C6—C7	118.92 (17)	O2—C16—C17	114.8 (2)
N1—C6—C5	112.52 (16)	C15—C16—C17	118.5 (2)
C7—C6—C5	128.56 (17)	C16—C17—H17A	109.5
C8—C7—C6	120.46 (18)	C16—C17—H17B	109.5
C8—C7—H7	119.8	H17A—C17—H17B	109.5
C6—C7—H7	119.8	C16—C17—H17C	109.5
C7—C8—C9	119.21 (19)	H17A—C17—H17C	109.5
C7—C8—H8	120.4	H17B—C17—H17C	109.5
C9—C8—H8	120.4		
			
C1^i^—Ir1—N1—C10	−89.11 (17)	C2—C1—C5—C4	−0.4 (3)
C1—Ir1—N1—C10	−176.28 (17)	Ir1—C1—C5—C4	178.33 (14)
O2—Ir1—N1—C10	91.72 (16)	C2—C1—C5—C6	−177.59 (17)
O2^i^—Ir1—N1—C10	3.15 (16)	Ir1—C1—C5—C6	1.2 (2)
C1^i^—Ir1—N1—C6	90.96 (14)	C10—N1—C6—C7	−3.9 (3)
C1—Ir1—N1—C6	3.79 (14)	Ir1—N1—C6—C7	176.01 (14)
O2—Ir1—N1—C6	−88.21 (14)	C10—N1—C6—C5	175.95 (17)
O2^i^—Ir1—N1—C6	−176.77 (14)	Ir1—N1—C6—C5	−4.1 (2)
C1^i^—Ir1—C1—C2	78.83 (17)	C4—C5—C6—N1	−175.00 (18)
N1^i^—Ir1—C1—C2	−1.26 (18)	C1—C5—C6—N1	1.9 (2)
N1—Ir1—C1—C2	176.04 (18)	C4—C5—C6—C7	4.9 (3)
O2—Ir1—C1—C2	−95.48 (17)	C1—C5—C6—C7	−178.21 (19)
C1^i^—Ir1—C1—C5	−99.77 (15)	N1—C6—C7—C8	2.4 (3)
N1^i^—Ir1—C1—C5	−179.86 (14)	C5—C6—C7—C8	−177.44 (19)
N1—Ir1—C1—C5	−2.56 (13)	C6—C7—C8—C9	1.0 (3)
O2—Ir1—C1—C5	85.92 (14)	C7—C8—C9—C10	−2.8 (3)
C5—C1—C2—C3	−0.6 (3)	C6—N1—C10—C9	2.1 (3)
Ir1—C1—C2—C3	−179.21 (15)	Ir1—N1—C10—C9	−177.85 (16)
C4—N2—C3—C2	0.1 (3)	C8—C9—C10—N1	1.4 (3)
C4—N2—C3—F1	−179.61 (17)	C4—O1—C11—C14	65.0 (3)
C1—C2—C3—N2	0.9 (3)	C4—O1—C11—C12	−60.2 (3)
C1—C2—C3—F1	−179.45 (17)	C4—O1—C11—C13	−177.8 (2)
C3—N2—C4—O1	176.95 (18)	C1—Ir1—O2—C16	−177.15 (16)
C3—N2—C4—C5	−1.3 (3)	N1^i^—Ir1—O2—C16	85.88 (16)
C11—O1—C4—N2	11.9 (3)	N1—Ir1—O2—C16	−96.84 (16)
C11—O1—C4—C5	−169.79 (18)	O2^i^—Ir1—O2—C16	−2.82 (13)
N2—C4—C5—C1	1.4 (3)	Ir1—O2—C16—C15	6.0 (3)
O1—C4—C5—C1	−176.82 (17)	Ir1—O2—C16—C17	−172.81 (14)
N2—C4—C5—C6	178.27 (18)	C16^i^—C15—C16—O2	−3.43 (16)
O1—C4—C5—C6	0.0 (3)	C16^i^—C15—C16—C17	175.3 (2)

Symmetry code: (i) −*x*, *y*, −*z*+3/2.

### Hydrogen-bond geometry (Å, º)

**Table:**

*D*—H···*A*	*D*—H	H···*A*	*D*···*A*	*D*—H···*A*
C7—H7···O1	0.95	2.27	2.870 (2)	120
C10—H10···O2^i^	0.95	2.48	3.089 (2)	122
C10—H10···F1^ii^	0.95	2.41	3.055 (2)	125
C12—H12*C*···N2	0.98	2.29	2.927 (3)	122
C14—H14*B*···N2	0.98	2.59	3.153 (3)	116

Symmetry codes: (i) −*x*, *y*, −*z*+3/2; (ii) *x*−1/2, *y*+1/2, *z*.

## References

[bb1] Brandenburg, K. (2005). *DIAMOND*. Crystal Impact GbR, Germany.

[bb2] Bruker (2006). *APEX2* and *SAINT*. Bruker AXS Inc., Madison, Wisconsin, USA.

[bb3] Frey, J., Curchod, B. F. E., Scopelliti, R., Tavernelli, I., Rothlisberger, U., Nazeeruddin, M. K. & Baranoff, E. (2014). *Dalton Trans.* **43**, 5667–5679. 10.1039/c3dt52739e 24345847

[bb4] Kang, Y., Chang, Y.-L., Lu, J.-S., Ko, S.-B., Rao, Y., Varlan, M., Lu, Z.-H. & Wang, S. (2013). *J. Mater. Chem. C*, **1**, 441–450.

[bb5] Lee, S. J., Park, K.-M., Yang, K. & Kang, Y. (2009). *Inorg. Chem.* **48**, 1030–1037. 10.1021/ic801643p 19166368

[bb6] Oh, H., Park, K.-M., Hwang, H., Oh, S., Lee, J. H., Lu, J.-S., Wang, S. & Kang, Y. (2013). *Organometallics*, **32**, 6427–6436.

[bb7] Park, J., Oh, H., Oh, S., Kim, J., Park, H. J., Kim, O. Y., Lee, J. Y. & Kang, Y. (2013). *Org. Electron.* **14**, 3228–3233.

[bb8] Sheldrick, G. M. (1996). *SADABS*. University of Göttingen, Germany.

[bb9] Sheldrick, G. M. (2008). *Acta Cryst.* A**64**, 112–122. 10.1107/S0108767307043930 18156677

[bb10] Westrip, S. P. (2010). *J. Appl. Cryst.* **43**, 920–925.

